# Relapse Model among Iranian Drug Users: A Qualitative Study

**Published:** 2015-01

**Authors:** Amir Jalali, Naiemeh Seyedfatemi, Hamid Peyrovi

**Affiliations:** 1 Department of Psychiatric Nursing, Nursing and Midwifery College, Substance Abuse Prevention Research Center, Kermanshah University of Medical Sciences, Kermanshah, Iran;; 2 Department of Mental Health Psychiatric Nursing, Nursing and Midwifery College, Iran University of Medical Sciences, Tehran, Iran;; 3 Department of Critical Care Nursing, Nursing and Midwifery College, Iran University of Medical Sciences, Tehran, Iran

**Keywords:** Drug Users, Grounded Theory, Iranian Drug User, Relapse Model

## Abstract

**Background: **Relapse is a common problem in drug user’s rehabilitation program and reported in all over the country. An in-depth study on patients’ experiences can be used for exploring the relapse process among drug users. Therefore, this study suggests a model for relapse process among Iranian drug users.

**Methods: **In this qualitative study with grounded theory approach, 22 participants with rich information about the phenomenon under the study were selected using purposive, snowball and theoretical sampling methods. After obtaining the informed consent, data were collected based on face-to-face, in-depth, semi-structured interviews. All interviews were analyzed in three stages of axial, selective and open coding methods.

**Results: **Nine main categories emerged, including avoiding of drugs, concerns about being accepted, family atmosphere, social conditions, mental challenge, self-management, self-deception, use and remorse and a main category, feeling of loss as the core variable. Mental challenge has two subcategories, evoking pleasure and craving. Relapse model is a dynamic and systematic process including from cycles of drug avoidance to remorse with a core variable as feeling of loss.

**Conclusion: ** Relapse process is a dynamic and systematic process that needs an effective control. Determining a relapse model as a clear process could be helpful in clinical sessions. Results of this research have depicted relapse process among Iranian drugs user by conceptual model.

## Introduction

Addiction and drug abuse are a social phenomenon in which individuals’ interaction plays an effective role.^1^ In many studies, individuals interact with each other and its role in therapy and even treatment failure has been emphasized.^2^ Hunter-Reel et al. (2009) argue that several individual and social environment factors affect the outcomes of treatment of drug abusers. Thus, the interaction between individuals and social factors has a very crucial role in treatment of such patients.^1^ Despite advances in treatment of alcoholism and the abuse of other drugs, frequent relapse and the lack of control on re-use of alcohol and other drugs are still a serious and essential problems in treatment of these patients.^3^ Westhuizen (2007) believes that relapse is a common phenomenon in drug users which the public considers as addiction treatment failure,^4^ while Roshani et al. argue that relapse is not only a failure in treatment, but also a situation that can affect  the client by re-using drugs even for one time of use.^5^ Therefore, relapse means returning to pre-treatment conditions,^6^^-^^9^ in which the drug user falls into a vicious cycle and his/her social, occupational, family, physical and mental characteristics are considerably reduced.^6^^,^^10^^,^^11^

Some factors have been discussed so far in scientific literature regarding identification of factors affecting the process of relapse, and post-treatment reuse of drugs.^12^^,^^13^ In this context, various models have been designed to depict such a phenomenon. Generally, there are two basic models. Psychological relapse models include Cognitive Behavioral Model by Marlatt et al., Pearson Situation Interaction Model by Litmann, Cognitive Appraisal Model by Sanchez-Craige, Self – Efficacy and Outcome Expectation Model by Wilson and Dynamic Developmental or Proposed Model by Marlatt and Witkiewitz. The psycho-biological relapse models include Opponent-Process and Acquired Motivation Model by Solomon, Craving and Loss of Control Model by Ludwig and Wikler, Urges and Craving Model by Wise, and Post-Acute Withdrawal Syndrome Model by Gorski and Miller.^13^ These models are all based on psychological status, situation and stress. Inferring the relapse process model that is based on social interaction and communication can be helpful in preventing relapse.^14^ Thus, studying the relapse process and proposing a model based social interactional is very helpful in rehabilitation programs since addiction is a social and human interactional problem.^1^

In relapse and drug-related research, qualitative studies are the best approaches due to their nature and human interactions, and interaction with the social environment plays important roles in the process. In qualitative research, the researcher can achieve the perspectives and factors influencing the life-cycle, and interactions.^1^ In addition, the researchers use the grounded theory method to understand human and environmental interactions, since the foundation of grounded theory research is based on symbolic interaction and interpersonal interactions; also, the grounded theory studies can lead to development of theory or model.^15^ In this research, we elaborated on the relapse process and determined a model for relapse behavior among drug users in Kermanshah city, the west of Iran. Therefore, the grounded theory methodology was used in this study to determine the relapse model in drug users.

## Materials and Method

*Study Design*

In this study, we needed to study the concept of relapse and required a holistic approach to understand how the relapse process occurs and which factors affect it. Thus, we selected a grounded theory method for this research which is a qualitative research approach used for the study of social processes in human interactions.^16^

*Setting and Participants*

In this study, the research environment was three public Drug Abuse Treatment Clinics and seven private clinics in Kermanshah, Iran. Participants were selected according to the study goals and their experience with the process of drug relapse. Initially, purposive sampling was applied and the people who had more experience of relapse (i.e. they had experienced relapse more than once, opiate consumer and agreed for participation in the study) were selected. Subsequently, they were first given the full information about the study in meetings with the individuals participating and they were asked to fill a consent form if they agreed to participate in the study after discussing the purpose and methods. In addition, they were asked to introduce other consumers of drugs they know with relapse experience in life and good information about drug abuse and relapse (snowball sampling). Two members of the client’s family and three addiction therapists who had a great experience in the field of addiction treatment and relapse were used as participants in this study. Purposive sampling followed by theoretical sampling by forming categories continued until data saturation. A total of 22 participants including 12individuals who were taking drug were selected based on purposive sampling and 10 participants were selected through theoretical sampling (five patients who were taking the drug, three family members and two therapists).

*Data Collection*

Data collection process began in February 2009 and continued until September 2011. Interviews with the participants were done individually at different times (morning and evening), and through meeting the participants at psychological clinic office. The main method of data collection in this research was in-depth interview using open-ended questions. Interviews were conducted for 30 to 70 minutes, recorded after the agreement of the participants, and then written as soon as possible. During the interview, it was also recorded so that factors such as tone of voice, pronunciation, laughter, crying and pause were also recorded. To conduct the interview and extract the concepts, thinking, attitudes, processes and perspectives of the participants for guiding the interview included questions such as: “What happened after you quitted smoking again?” and “how about your feelings about giving up after cessation of drug abuse and reuse?” During the interview, the participants answered the questions and were more persuaded to clarify the details of their response. A total of 25 sessions were conducted for individual interviews of 22 participants.

*Data Analysis*

In order to study the relationship between codes and concepts, as well as the constant comparative data analysis to explore the relationships between categories and to obtain the model, the analysis of Strauss and Corbin (1998) was used. In this study, the interviewers carefully listened to the recorded interviews. After listening to each interview for several times, the researchers wrote them down. At this stage, all the interviews were carefully written, the written interviews were read, and re-open coding was carefully done. In the open source code, a total of 1057 initial codes were obtained which formed 23 categories. After open coding, axial coding was attempted. Therefore, the code in the open coding of the interviews was compared and similar codes were placed in a group. The data were compared with the initial categories regularly and the first 23 categories of the main level dropped to nine.

*Trustworthiness*

Accuracy determined by four criteria of credibility, conformability, dependability, and transferability. The four criteria are known as the four criteria of Guba and Lincoln (1985), and are acceptable for determining the accuracy of qualitative research.^17^ For credibility of the results, the researcher used interviews and field notes to collect the data. Long-term attendance in the clinical setting, long-term engagement with data, and experience as a psychiatric nurse and treatment team for over five years helped the researchers combine data and gain a deeper understanding of the participants’ experiences. After open coding and interviews, two-group sessions of 1.5-hour interviews with 11 people (a group of 6 and a group of 5 people) were organized and the resulting codes were discussed and checked by interviewees. For further research, three participants were contacted to further explain certain subjects, each holding a 20-minute session. For credibility of the results, the researcher in this study tried to fully express all the research in writing so that other researchers are able to track the data. In addition, the researchers have tried to accurately describe all the steps, participants, track and field studies so as the readers should be able to fit the application in the context of their situation and can easily check everything. This research was approved in Iran University of Medical Sciences ethics committee in 2009.

## Results

Participants’ demographic characteristics are shown in Table 1. Nine categories and one main category were obtained and made clear by optional coding among categories and the core category. Categories and sub-categories of the study are presented in Table 2.

**Table 1:** Characteristics of the participants (clients)

**Characteristics**	**n=17**
Gender, %(n)	
Male	70.59 (12)
Female	29.41 (5)
Age in years	
Median, range	29, 22-45
Education	
<high school	17.65 (3)
High school diploma	52.94 (9)
College or above	29.41 (5)
Marital status	
Single	52.94 (9)
Married	29.41 (5)
Divorced	17.65 (3)
Opiate use in years	
Median, range	6.5, 3-22
The frequency of relapse	
Median, range	3, 1-4

**Table 2:** Category and subcategory of relapse process among Iranians

**Main category**	**Category**	**Sub category**
Feeling of loss	Avoiding drugs	Physical Unhealthy
		Nutritional Disturbances
		Sexual Problems
		Susceptible to use
		Behavioral Changes
	Concern about being accepted	Needs to being confirmed
		Needs to being Accepted
Family Atmosphere	Structure
	Attitude
	Tension
Social condition	Interaction with addict people
	Easy access to opiates
	Social environmental pressure
Evokes pleasures	Dream of opiate
	Embodiment of drug use
Craving	Worry and distress
	Mental challenge
Self-Management	Ability or Strength
	Behavioral management
Self-Deception	Rationalization
	Behavior reactions
Use	Individual indicators
	Interaction indicators
Remorse	Excitement indicators
	Behavioral indicators

*Model*

In the relapse model resulting from the research findings, the main process begins with the step of avoiding drugs. In the drugs avoidance stage, the patient experiences the withdrawal symptoms. At this stage, the patient always deals with the effects of family atmosphere, social conditions as well as feeling of scarcity and deficiency in addition to drugs avoidance consequences. Meanwhile, he/she is also concerned with the challenge of being accepted. The patient tries to remember the enjoyment of drug abuse and the sweet moments of using drugs to get rid of such a situation. At the same time, self-management and self-deception mechanisms are also formed in the patient’s mind. However, such mechanisms are not still highlighted due to dreaming and  evoking pleasure the nature of the sweet pleasures of drug abuse. 

The pleasure and dreaming of drug use leads to craving in the patient; here, self-management and self-deception mechanisms become stronger and some challenges occur in the patient’s mind. For using drugs, effects of family environment and social conditions, feeling of loss and concern with being accepted associated with two mechanisms of self-management and self-deception cause a dilemma of using or not using the drugs for the patient.  If the effects of family environment and social conditions can have positive effects on the patient, and the patient can achieve self-management mechanism using his previous experiences and psychological reactions, he will persist in avoiding drugs and will not abuse drugs. If the effects of family environment stimulate the patient to use drugs and the mechanisms of self-deception dominate based on making reasons, the patient tends towards drugs and will use them. 

**Figure 1:**
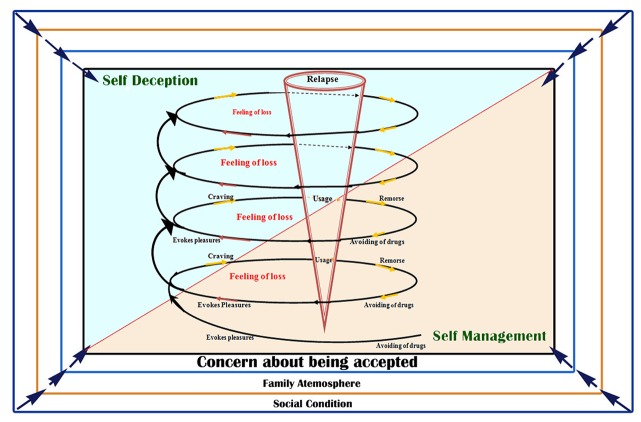
Iranian relapse model

After using drug and loss of euphoria effects, the patient realizes his reality and becomes regretful, ashamed and feels guilty and decides to quit using drugs for ever. Then, the patient experiences the avoidance of drugs stage again and the cycle occurs again. The distance between avoiding drugs and regretfulness is called a lapse and due to lowering the distance between lapses, the patient will be forced to use drugs when the relapse has occurred. As can be seen, in this cumulative model, which is like a standing spring, the relapse process means gathering of few cycles that start with avoidance of drugs stage and leads to repentance (Figure 1). Here, the concepts in two stages and the relationships between them will be interpreted.

*Concepts of the Model*

- The concept of social conditions
  The model is placed on four contexts or levels. The first context is social conditions that work as the underlying factors in this model. Social conditions include interactions with addicts, easy access to drugs, and environmental and social pressures. Social conditions are a variable which interacts with all components of the process and can be effective on relapse process by influencing the family atmosphere, loss and the concern of being accepted.
- The concept of family atmosphere
  This is also one of the main variables of the model affected by social conditions and interacts with all components of the process. This concept consists of attitude, interaction and structure. The family atmosphere can have a dual effect on the relapse process. If the family has stability in its structure, and the attitudes are logical and positive with constructive interactions established in the family, the family atmosphere can have a positive effect on the patient and assist him/her in avoiding drugs. Otherwise, the family atmosphere would be itself a factor leading the patient to abuse the drug.
- Concern about being accepted
  This concept is an intellectual and effective factor in the relapse process, which is always present from the beginning of the drugs avoidance – remorse cycle; however, its presence and effect can be more or less due to social conditions, family atmosphere, and other variables affecting the patient. But, such a concern in mind becomes much stronger at the time of craving for drugs as well as at the end of the cycle while remorse occurs. The concept includes the need for approval and the need for acceptance. As can be seen in the model, this variable is affected by social conditions and family atmosphere variables and interacts with all components of the process.
- Feeling of loss
  The concept of feeling of loss appears in this theory as the core variable. Since this class was associated with other classes, it was frequently seen in the whole process. The patients had always the feeling of loss since withdrawal and abstinence from the drugs; such a feeling of loss caused distress or stress of loss for them. This class is related to all components of the model.
- Self-management and self-deception mechanisms
  These mechanisms involve the patient’s mind actively since the onset of association until the remorse time and it causes psychological reactions by the patient. This mechanism plays an important role in the relapse model. The self-management and self-deception mechanisms play their own opposite roles in the relapse model. As can be seen in linear and cumulative models of relapse process, if the self-management mechanism has a strong role, it will result in drug abstinence; in contrast, the individual is led to laps, and ultimately to relapse by dominance of self-deception. The self-management and self-deception mechanisms are shown as two triangles, which complement each other.
- Avoidance of  drugs
  Avoidance of drugs is a concept that begins with the drugs abstinence-remorse cycle. This cycle is called as the lapse’s cycle. Abstinence from drugs is a variable that contains physical illness, nutritional problems, sexual problems, vulnerability to drug abuse and behavioral changes subcategories. Avoiding drugs is a factor associated with family atmosphere, social conditions, concern about acceptance, feeling of loss,  and self-management and self-deception mechanisms. This variable is shown as the antecedent of association concept in the model.
- Evoking pleasure
  The evoke pleasure notion is a subjective variable used by the patient in order to avoid problems or recall previous good and enjoyable memories. Evoke pleasure occurs in the patient’s mind during failure, psychological stresses, happiness, and especially in loneliness. Association is itself a subcategory of mental struggling, which as an important variable in the relapse process that interacts with variables of family atmosphere, social conditions, feeling of loss, abstinence from drugs and self-management and self-deception mechanisms and acts as the antecedent of craving concept.
- Craving
  Craving is also a variable that belongs to the mental challenge subcategory. Craving is a very strong inner and mental strength causing anxiety and apprehension for the patient and leads him to drug abuse. Craving interacts with the variables of family atmosphere, social conditions, concern about being accepted, and feeling of loss and evoke pleasure. The most important variables associated with craving are self-management and self-deception mechanisms formed in response to craving. Craving, itself, plays an important role in the relapse process as the antecedent of drug abuse.
- Use
  The concept of use is a variable consisting of two individual and interactive components in which the syndromes of use individually and interactively occur. Use also interacts with the variables of craving, family atmosphere, social conditions, concerns about being accepted, feeling of loss, self-management and self-deception mechanisms, and plays an important role as the antecedent of remorse.
- Remorse
  The concept of remorse is the last part of the lapse’s cycle. This variable also includes two components of emotional and behavioral markers. It interacts with variables of family atmosphere, social conditions, and concerns about being accepted, feeling of loss and self-management and self-deception mechanisms and can act as an antecedent for the variable of avoidance of drugs.

*Relapse Model Characteristics*

- In this model, the variables including social conditions, family atmosphere, and concerns about being acceptance and self-management and self-deception mechanisms interact with each other and there is no definite boundary between them. For this reason, they are separated from each other in the model with dotted lines. In this model, the variables are influenced  in the process; however, their effects are not the same at every point and at any time of the model, and the severity of effects may vary according to the patient’s condition.
- Family atmosphere and social conditions are associated with other components as the underlying factors in this model.
- Self-management and self-deception mechanisms are in conflict with each other, and by increased effect of self-deception, the relapse rate increases and self-management effects are reduced.
- Each of the avoiding of drugs–remorse cycles are called a lapse, which starts with abstaining from drugs, leading to reminiscence of sweet moments, craving for drug abuse, drug abuse and eventually remorse. The intervals between these cycles represent the period which are not identical and can vary from few hours to few days, months or even more. The patient can be directed toward relapse and re-use of drugs through each of these cycles. With increase in the number of lapses (avoiding of drugs - remorse cycles), the interval between them is less; thus, the probability of relapse is more than the past.  

## Discussion

The results showed that relapse model consisted of cycle or cycles of avoiding of drugs - remorse. The relapse process in this model starts with avoiding of drugs and the next cycle begins by reaching remorse. However, it is possible for the patient to move toward relapse from drug use and another cycle will not develop. The model was the result of review of social processes and interactions; it was obtained through study and exploration of experiences of drug users who had themselves experienced relapse. In this model, like psychological relapse models, the physical factor, which is one of the similar physical symptoms of withdrawal, is associated with contextual factors and cognitive mechanisms may show more prominent effects and can become grounds for the relapse.^14^

The model of relapse in this study draws a dynamic process, in which the stages interact with each other, and a common boundary cannot be distinguished between each of the variables or processes.  In the dynamic developmental model,^14^ more emphasis  is also on the dynamics of situations rather than expansion of them. This is a developed model of the prevention model of Marlatt and Gordon’s cognitive-behavioral relapse. In cognitive-behavioral relapse model,^14^^,^^18^ the interaction with the underlying factors, physical condition as decreased body weight, cachexia and body numbness, sexual disturbance as premature ejaculation and loss of libido,^18^ cognitive processes, motivation and coping skills has been emphasized.^19^

Core variable in the relapse model obtained from the results of the present study is the feeling of loss. The results showed that the feeling of loss was present at all stages of relapse process and is associated with all of the model variables and concepts. Core variable is not seen in the dynamic developmental or proposed model of relapse, and the proposed model consists of three main parts of tonic processes, phasic responses and conceptual factors.^14^

The study results showed that self-management and self-deception mechanisms were two mental mechanisms existing in the relapse model, and with overcoming each one of them, the other one becomes more ineffective. These mechanisms include a behavioral component that may be seen by their increased effects in the relapse model. The self-management mechanism consists of two parts of ability and behavioral indexes which are developed to control the situation and create compatibility reactions in order to remain in a state of abstinence from drugs by increasing their effects. The self-deception mechanism also consists of two parts of alleging and behavioral symptoms. This mechanism helps the patient sooth himself for using drugs, as well as making his drug abuse reasonable for him and others. By dominating this mechanism, the craving effects for drugs increase and the motivation for drug abuse are elevated, and the patient tends to use drug for calming himself and will use drugs. There are also cognitive processes in the dynamic developmental or proposed model of relapse that are similar to self-management mechanism in some cases. In this model, there is the concept of self-efficacy that can cause compatibility responses in the patient and put him away from the drugs. There is also craving in cognitive processes that makes the patient prone to the behavior of drug abuse and leads to drug reuse associated with physical deprivation, inappropriate emotional state and lack of individual ability to develop adaptation responses.^12^^,^^14^

The results showed that relapse or lapse could result from one or more cycles of avoiding of drugs-remorse. In this cycle, there were drugs avoidance, remembrance of sweet moments of drug abuse, craving for drug abuse, drug use and remorse and the patient seemed to be calm at first by using drugs; but after a while and becoming aware of his status, he would experience the feeling of disgust and depression, shame and remorse. In the dynamic developmental or proposed model of relapse, there are also concepts such as craving, motivation for use, cognitive processes, behavior of drug abuse(in terms of quantity and behavior frequency).^12^^,^^20^

This study was presented as a model of the relapse process. In this model, various stages of the process are repeated one after another and finally it could lead to relapse after the abuse variable in the first cycle or the next cycles any of which is called slip. As the lapse increases, the distance between them is reduced and a greater propensity to drug abuse and relapse is also more likely to happen.

In this model, the role of family atmosphere appeared to be highly significant. According to the Iranian customs and culture, family is the most important element and factor in individuals’ personality and interactions. Thus, the family's role in the process and rehabilitation of patients, especially taking drugs by the patients is irrefutable. The importance of family was also highly emphasized in patients’ interviews, and the study results also confirmed such a finding. In contrast, the role of family in different models and studies in the western countries had not been observed so evidently as this study. Some studies have briefly suggested the family and its role(14). In Iranian family, the mentioned factors were highlighted and can have further positive and negative effects on the treatment process of the patients because of the deep emotional connections and unique cultural context in the western regions of the country where the family always supports the individual and considers itself involved in his problems. Thus, adequate training for the families can make their responses, either verbal or emotional, more decent and prevents the exposure of the patient to relapse.

Only five female drug users participated in the study, because many of them were not willing to participate in the study for family reasons.  In addition, many of drug users (men and women) did not want to record their interviews and we selected the participants with difficulty. 

## Conclusion

It can be concluded from the results of the study that patients have the nostalgic feeling or feeling of losing something during recovery time and withdrawal, and due to internal desire to take drugs, they seek to find a reason or solution to resume the drug use. In addition, family’s economic problems, distance from family, and inappropriate family conflicts can make great psychological and intellectual problems for patients who are aware of their condition and prepare themselves for continual treatment.  Also, continued problems and inappropriate behaviors of family and relatives as well as family mistrust and several doubts of families can cause physical and mental fatigue in patient; thus, the patient finds the only way to escape from problems by using drug. Finally, it can be stated that this model is the result of the patients’ experiences of relapse or relapse process that is interpreted from their speech. By studying the components of this model, appropriate information can be obtained for a more detailed and more suitable planning for preventing relapse in patients taking drugs.

This model could help the clients and their families, therapists, social workers and other clinical specialists. Results of this research showed the relapse process step by step; thus, clinicians could plan for prevention of relapse. Researchers suggest that quantitative research should be done for each effective variable on the model. 

## References

[1] McDonnell A, Van Hout MC (2010). A grounded theory of detoxification-seeking among heroin users in south east Ireland.

[2] Kenny K (2007). A grounded theory model of the counselling referral process in primary care methadone treatment in Ireland [thesis].

[3] Mckay JR, Rutherford MJ, Cacciola AS, Cacciola JS (1996). Development of the Cocaine Relapse Interview: an initial report. Addiction.

[4] Westhuizen VD, Marichen A (2007). Exploring the experience of chemically addicted adolescents regarding relapse after treatment [thesis].

[5] Roshani B, Jalali A, Bidhendi SH (2014). Study the causes of relapse among Iranian drugs users in Kermanshah. Life Science Journal.

[6] Bain KA (2004). Exploring the subjective psychological experience of relapse in cocaine/crack and heroin users [thesis].

[7] Fisher GL, Nancy RA (2009). Encyclopedia of substance abuse prevention, treatment and recovery.

[8] Grusser SM, Thalemann CN, Platz W (2006). New approach to preventing relapse in opiate addicts: A psychometric evaluation. Biological Psychology.

[9] Levy MS (2008). Listening to our clients: The prevention of relapse. Journal of Psychoactive Drugs.

[10] Allosp S, Saunders B, Phillips M (2000). The process of relapse in severely dependent male problem drinkers. Addiction.

[11] Brown TG, Seraganian P, Tremblay J, Annis H (2002). Process and outcome changes with relapse prevention versus 12-Step aftercare programs for substance abusers. Addiction.

[12] Ramo DE (2008). Developmental Models of Substance Abuse Relapse [thesis].

[13] Connors GJ, Maisto SA, Donovan DM (1996). Conceptualizations of relapse: a summary of psychological and psychobiological models. Addiction.

[14] Witkiewitz K, Marlatt A (2004). Relapse prevention for alcohol and drug problems: That was Zen, This is Tao. American Psychologist.

[15] Larossa R (2005). Grounded theory methods and qualitative family research. Journal of Marriage and Family.

[16] Corbin J, Strauss A (2008). Basics of qualitative research: techniques and procedures for developing grounded theory.

[17] Holloway I (2005). Qualitative research in health care.

[18] Seyedfatemi N, Peyrovi H, Jalali A (2013). Sexual activity in relapse process among Iranian drug users- A qualitative study. Life Science Journal.

[19] Miller WR, Westerberg VS, Harris RJ, Tonigan JS (1996). What predicts relapse? Prospective testing of antecedent models. Addiction.

[20] Seyedfatemi N, Peyrovi H, Jalali A (2014). Relapsing experience in Iranian opiate users: a qualitative study. Int J Community Based Nurs Midwifery.

